# Impact of syllabic constraints on error production in Korean speech: a study of serial order control with practice

**DOI:** 10.3389/fpsyg.2026.1612386

**Published:** 2026-03-13

**Authors:** Sangyub Kim, Satoru Saito, Kichun Nam

**Affiliations:** 1 Department of Psychology, Chonnam National University, Gwangju, Republic of Korea; 2 Graduate School of Education, Kyoto University, Kyoto, Japan; 3 School of Psychology, Korea University, Seoul, Republic of Korea

**Keywords:** speech error, speech error induction technique, syllabic constraint, syllable, transposition error

## Abstract

This study tested whether serial order in Korean speech production is shaped by a language-specific, hierarchical syllabic organization. Using a speech-error induction paradigm, native Korean speakers produced four-syllable CVC nonwords while hearing auditory nonword distractors that targeted specific within-syllable positions (onset, vowel, coda). We hypothesized tighter coupling of onset–vowel (CV/body) than vowel–coda, predicting greater distractor-induced errors at codas. The results supported this prediction, with coda positions showing reliably higher error rates than onsets and position-specific memory effects concentrated in coda slots, indicating an interaction between syllabic status and serial memory. With repetition, errors declined selectively for onset–vowel pairings, whereas coda vulnerability persisted, showing that practice strengthens serial stability primarily where the hierarchical linkage is already strong. Together, these findings demonstrate that the ordering of segments in Korean is not governed by a flat, segment-by-segment sequence; rather, it reflects a language-specific hierarchy in which CV/body functions as a more cohesive unit than VC. The finding thus provides convergent evidence that serial order control in Korean is constrained by its syllabic architecture, with implications for cross-linguistic models of phonological encoding and for theories that integrate hierarchical structure into serial planning mechanisms.

## Introduction

In daily communication, speech is a crucial aspect of language production that remains incompletely understood. Here we examine the cognitive processes that coordinate sequential planning with the hierarchical structure of phonology, asking how temporal information is encoded during spoken word production. Following Lashley (1951), we treat serial order not as a flat left-to-right listing of units but as behavior constrained by a multi-level organization in which higher-order structures gate lower-level sequencing. In speech, these structures include language-specific planning units that shape how phonemes are assembled into syllables and how syllables are staged across time (Acheson and MacDonald, 2009a; Botvinick and Plaut, 2006; Brown et al., 2000; Guitard and Cowan, 2023). Phonological encoding specifies the ordering of subsyllabic elements for articulation, but this ordering is conditioned by a language’s preferred syllabic organization. Cross-linguistic work shows that the functional unit of planning varies—e.g., onset-rhyme in English, mora in Japanese, and body–coda organization in Korean (Hooper, 1972; Selkirk, 1982; Vousden et al., 2000; Nakayama and Saito, 2014; Yi, 1998)—implying that what looks like a “serial” phenomenon often reflects language-specific hierarchical structure. Accordingly, we ask whether Korean’s syllabic organization modulates serial order control during production.

Serial order control is crucial for fluent and coherent communication, as it coordinates the timing and arrangement of various linguistic components during the act of speaking. Serial order control is an essential cognitive process that interacts with physical features such as phoneme positions within a word, as well as phonological structure properties (Acheson and MacDonald, 2009a; Brown et al., 2000; Botvinick and Plaut, 2006; Lashley, 1951). Phonological encoding, which specifies the sequencing of syllables and phonemes within a syllable for articulation, is critical to this process. The syllable, as the fundamental unit within a word, plays a pivotal role in phonology (Hooper, 1972; Selkirk, 1982), and is subject to natural phonological processes, which hold efficiency in a given language (Content et al., 2001). This syllabic efficiency is achieved under specific constraints unique to each language during speech, such as the onset and rhyme structure in English (Vousden et al., 2000), morae regularities in Japanese (Nakayama and Saito, 2014), and the body and coda composition in Korean (Yi, 1998). The differences in syllabic constraints between languages suggest that there can also be variation in serial order control between languages, potentially affecting temporal representation of serial order memory in speech production. Therefore, investigating the link between syllabic constraints and serial ordering in speech in a variety of languages is crucial for understanding general principles in speech production. In this study, we examined whether the structural constraints of a syllable influence the phonological encoding governed by cognitive serial order control, using a sequence of Korean syllables for which studies have been underrepresented thus far.

### Main principles of serial order control

The present study investigated the relationship between serial order control and four principles related to serial memory in speech production. These principles include the phonological similarity principle, temporal distance principle, edge principle, and syllabic constraint principle, each offering a distinct perspective on how serial memory functions in speech production. The phonological similarity principle suggests that phonemes or syllables that sound similar are more likely to be confused with one another during speech production. The phonological similarity effect is well-documented in memory studies (Acheson and MacDonald, 2009a; Saito and Baddeley, 2004; Page et al., 2007), where items that sound alike are often more difficult to recall in the correct order than dissimilar items. In speech production, this principle means that errors are more likely when trying to produce phonologically similar sequences, as they compete for activation (Dell, 1986). This leads to increased instances of substitutions, deletions, or transpositions of sounds, particularly when the speaker is trying to articulate complex or unfamiliar word sequences.

Furthermore, according to the temporal distance principle (e.g., Botvinick and Plaut, 2006), elements that are close in temporal sequence are more susceptible to interference and confusion. In speech production, this principle predicts that phonological units occurring in close temporal proximity are more likely to interact, leading to increased exchange or misordering errors. For example, when comparing 1st–2nd and 1st–3rd positional exchanges, the principle predicts a higher error rate for 1st–2nd exchanges because the two syllables are temporally adjacent, increasing the likelihood of interference between their serial representations. This pattern reflects the idea that the temporal distance between elements constrains their degree of mutual activation, such that closer elements compete more strongly during sequencing. However, cross-linguistic evidence for this principle varies depending on the phonological organization of each language. In English, within-word exchange errors are rarely observed despite the temporal adjacency of phonemes, a pattern attributed to strong syllabic constraints that tightly bind internal phonological units. In contrast, Japanese, whose phonological structure is organized around morae rather than syllables, shows within-word exchange errors that align with the predictions of the temporal distance principle (Nakayama and Saito, 2014).

The edge principle is grounded in the observation that items at the beginning and end of a sequence are remembered better than those in the middle, known as the primacy and recency effects, respectively (Gupta, 2005; Gupta and Tisdale, 2009a). In speech production, this principle suggests that the phonological elements at the edges of words or phrases are more robustly encoded and thus less susceptible to errors. This enhanced encoding at the boundaries can be attributed to increased cognitive emphasis on the initiation and termination of speech, which are critical for maintaining the integrity and intelligibility of spoken language. The syllabic constraint principle focuses on the structural constraints of syllables, particularly noting that elements occupying the same syllabic position (e.g., all onsets or all codas) tend to be more similarly represented and processed (Vousden et al., 2000). In speech production, this can mean that errors are more likely to occur between similar positions across different syllables, such as exchanging onsets with onsets. For example, the ease of exchanging onset consonants across syllables suggests a patterned, position-dependent processing strategy that facilitates phonological planning but also predisposes types of errors under complex conditions. In applying these principles to Korean nonwords, particularly considering the CVC structure in Korean syllables, the current study aimed to clarify how these theoretical constructs affect the actual mechanics of speech production in a language that has not been extensively studied in this context. By using nonwords to minimize lexical familiarity, we test how three well-established regularities of ordered recall—phonological similarity, edge, and syllabic (position) structure—emerge when serial selection is embedded within a Korean-specific syllabic hierarchy, consistent with accounts in which predictive monitoring progressively sharpens serial representations under interference (Teghipco et al., 2023).

### Verbal working memory and its role in speech production

Verbal working memory plays a foundational role in both language comprehension and speech production. It serves as a specialized short-term system for the temporary storage and active rehearsal of sound-based information, maintaining the sequential integrity of phonological representations during speech planning and articulation (Baddeley, 2003). Verbal working memory is assumed to be composed of two interacting subsystems: One is closely related to speech perception system, which retains auditory-verbal information for a brief duration, another is based on speech production system, which refreshes this information through subvocal rehearsal to prevent decay (Baddeley, 2000; Baddeley et al., 1998). Together, these components enable speakers to sustain and manipulate phonological sequences long enough to assemble them into coherent speech output.

In the context of speech production, verbal working memory functions as a temporary buffer for sequencing phonological units such as phonemes, syllables, and words, ensuring that they are produced in the correct temporal order (Page and Norris, 1998; Gupta and MacWhinney, 1997). It supports the serial ordering process by maintaining activation gradients that represent the temporal positions of items in a sequence (Botvinick and Plaut, 2006). This mechanism allows the speech system to select and execute each phonological element in its proper order, while minimizing interference from adjacent or similar elements. When auditory distractors are presented, they enter the working memory system through the speech perception part of the system. If distractor presentation is immediately before producing speech sounds and the distractor is phonologically similar to the speech sounds, competition within verbal working memory intensifies, often leading to substitution, omission, or transposition errors (Saito and Baddeley, 2004). Such disruptions reveal the role of verbal working memory in coordinating the timing of phonological retrieval and articulatory execution, particularly under conditions of interference or repetition.

Importantly, in the present study, models of verbal working memory provide the theoretical foundation for understanding how serial order control operates during Korean nonword production. Because Korean syllables possess a clear CVC (Consonant–Vowel–Consonant) structure, they offer an ideal framework to examine how verbal working memory manages the sequential activation of subsyllabic units (onsets, nuclei, codas) within a language that lacks ambisyllabicity. In this study, phonologically similar versus dissimilar distractors are used to test how competition within verbal working memory modulates speech accuracy, revealing how serial order maintenance interacts with phonological similarity and syllabic constraint.

### The exploration of speech error pattern by speech error induction technique

In exploring the complexities of speech production, the current study adopts the speech error induction technique originally developed by Saito and Baddeley (2004), which is instrumental in probing the dynamics of serial order control within nonwords. This methodology employs the potential of phonological distractors to induce speech errors, thereby providing a unique lens through which to examine the cognitive processes underlying speech production. The technique involves presenting participants with phonological distractors immediately before their target utterances. This setup is designed to induce errors in speech production, thereby revealing insights into the mechanisms of serial ordering. In this setup, two types of distractors were used. The first distractor was phonologically similar distractors. These distractors share phonetic or phonological properties with the target sequences. They are anticipated to be more effective in inducing errors due to their closeness in sound to the target, which increases the likelihood of confusion and erroneous production. The second distractor was phonologically dissimilar distractors. These are less similar to the target sequences and are expected to induce fewer errors, serving as a control to highlight the effects of phonological similarity.

Verbal working memory is critical for the temporary storage and manipulation of phonological information (Baddeley and Hitch, 1994). It plays a vital role in speech production by maintaining phonological data within an accessible buffer. When distractor sequences are introduced, they compete with the target sequences for the same phonological buffer within verbal working memory. This competition can be stronger when the distractors and the targets are phonologically similar as they share a lot of phonemes making it difficult to clearly distinguish them, leading to an increased frequency of speech errors such as phoneme substitutions or deletions.

By utilizing this speech error induction technique, the current study aimed to examine how the serial order of phonological elements is managed within the framework of verbal working memory. Analyzing the errors induced by phonologically similar versus dissimilar distractors are expected to present insights into the cognitive processes of speech planning and execution.

### Syllabic constraint effects on the serial order control in speech production

The exploration of serial order control in speech production has been a significant area of research across languages, each presenting unique linguistic characteristics that influence the understanding of this cognitive process. Notably, studies conducted in languages like English, Japanese, and Korean provide insights into how phonological structures and syllable boundaries affect serial order control.

In English, the phonological marking of syllable boundaries has been a subject of extensive research (e.g., Blevins, 1995). However, the concept of ambisyllabicity introduces a complexity in English phonology. Ambisyllabicity refers to a phenomenon where a consonant at the end of one syllable also serves as the onset of the next syllable (Treiman and Danis, 1988; Kahn, 1980). This overlap can create challenges in identifying clear syllable boundaries, complicating the study of serial order control as it may affect how phonological sequences are processed and produced. The occurrence of ambisyllabicity may lead to variations in speech production strategies, potentially influencing error patterns and phonological planning. In addition, Japanese language studies often focus on the roles of both syllables and morae (Nakayama and Saito, 2014). A mora is a phonological unit that is smaller than a syllable but plays a critical role in the timing and rhythm of Japanese speech. The importance of morae alongside syllables adds a layer of complexity to the examination of serial order control in Japanese. This is because both units must be considered when analyzing speech errors and phonological organization, providing a richer context for understanding the interactions between subsyllabic units and larger linguistic structures.

However, Korean offers a distinct perspective due to its clear and well-defined syllabic structure, free from the complexities of ambisyllabicity (Yi, 1998; Han and Lee, 2003; Kim and Seok, 2006). Each Korean syllable is rigidly structured into onset, nucleus, and coda. Korean syllables obey tight phonotactic constraints that delimit what can occur in each slot and how adjacent slots interact. Onsets are single consonants (native clusters are not permitted) and include plain, tense, and aspirated obstruents as well as sonorants. A null onset is also licit in Korean and is represented orthographically by the placeholder consonant “ㅇ” (e.g., 아 /a/). Certain consonants never occur in onset (*ŋ*), and tense series (ㅃ, ㄸ, ㅉ) are restricted to onset position. Nuclei are monophthongs (아 /a/, 어 /ʌ/, 오 /o/, 우 /u/, 으 /ɯ/, 이 /i/) and a limited set of vowel–glide combinations (e.g., 왜 /wɛ/, 위 /wi/). Codas (“batchim”) are phonemically wide but surface-neutralize to seven categories /p, t, k, m, n, ŋ, l/ with unreleased obstruents; thus spelling-final ^ㅂ, ㅍ → /p¬/; ㄷ, ㅌ, ㅅ, ㅆ, ㅈ, ㅊ, ㅎ → /t¬/; ㄱ, ㅋ, ㄲ → /k¬/ (e.g., 앞 /ap¬/, 옷 /ot¬/, 밖 /pak¬/). Because of this inventory and the lack of onset clusters, the set of permissible C1–V1–C2 combinations is highly constrained. Any legal onset can combine with any nucleus, but C2 must map to one of the seven coda classes, and no voicing or laryngeal contrasts are realized word-finally.

These categorical restrictions interact with well-attested alternations across syllable boundaries, which are exactly the kinds of processes that can differentially bind the onset–vowel (“body”) vs. the vowel–coda juncture. Liaison/resyllabification shifts a coda to the following onset before a vowel, often de-neutralizing its place features (밖에 /pak + e/ → [pa.ke]; 국어 /kuk + ʌ/ → [ku.gʌ]). Nasal assimilation changes a coda obstruent to a homorganic nasal before a nasal onset (앞말 /ap¬+mal/ → [am.mal]). Liquid assimilation yields /nl/ → [ll] and /ln/ → [ll] sequences (신라 /sin+la/ → [실라 sil.la]). Tensification fortifies a following onset after certain codas or morphemes (밥거리 /pap¬+kʌri/ → [pap¬.k¬ʌ.ri]). Palatalization affects coronal segments before /i, j/ (디 /ti/ → [tɕi], 같이 /katʰ+i/ → [ka.tɕʰi]). Note that these alternations typically reconfigure the status of the coda or the following onset while leaving the onset–vowel pairing within the source syllable intact, which makes the onset–nucleus (“body”) cohesion more stable than the nucleus–coda linkage. This asymmetry provides a phonological rationale for expecting greater susceptibility to interference at codas than at onsets in our nonword task, and it aligns with evidence that Korean planning often recruits a CV-body-like unit more strongly than a coda-anchored unit.

This clarity allows for a more straightforward examination of how syllabic constraints influence serial order control. The hierarchical organization of Korean syllables enables precise investigations into how each component—onset, nucleus, and coda—contributes to the formation and execution of speech sequences. Converging psycholinguistic data further suggest that Korean recruits a relatively coarse proximate unit during planning—often at the syllable or CV-body level—implicating stronger onset–vowel cohesion than nucleus–coda (Verdonschot et al., 2021; Li et al., 2022). This structural clarity is particularly advantageous for studying the mechanisms underlying phonological processing and error occurrence in speech production.

### Examination of speech error patterns with nonword speech production

Using nonwords in the current study of serial order control in speech production offers four distinct advantages, particularly when examining the underlying linguistic and cognitive processes involved. The first advantage is circumventing the issue of lexicality. In Korean, changing syllable elements such as the onset can transform recognizable words into nonwords. This transformation is critical because it helps to reduce the influence of lexical familiarity, which can confound the effects being studied. Lexical familiarity refers to the ease of processing words that are frequently encountered and well-known by the speakers. By using nonwords, researchers can more effectively isolate the phonological and articulatory processes involved in speech production without the involvement of lexical retrieval processes, which might mask the pure effects of serial order control mechanisms. This approach ensures that the focus remains on how syllables and sounds are organized and produced, rather than on retrieving and processing known words. The second advantage is examining the impact of speech practices on serial order control. Nonwords, by virtue of having no prior usage or history, provide a unique tool to explore how new or unfamiliar phonetic sequences are handled by the speech production system. This is particularly useful for understanding how speakers deal with novel sequences and apply phonological rules to these sequences. Since nonwords do not come with pre-existing pronunciation patterns or biases, they can help reveal the fundamental processes and constraints involved in the serial ordering of sounds, independent of past experience and habitual usage. The third advantage is creating perfect CVC-structured syllables. Nonwords allow researchers to design stimuli that strictly adhere to the CVC (Consonant-Vowel-Consonant) structure, which is prevalent in many Korean syllables but not universally so across all words. By using nonwords that conform exactly to this structure, studies can eliminate variability in syllable structure that may exist in natural words, which can sometimes introduce inconsistencies between experimental conditions. This controlled approach ensures that the variables under investigation are not confounded by structural irregularities, making it easier to interpret the effects of syllable structure on speech production processes. The final advantage is investigating serial order control in syllables. The use of nonwords facilitates a more detailed investigation of how individual parts of the syllable-onset, nucleus, and coda-are controlled during speech production. Since nonwords can be tailored to target specific structures or sequences, they provide a precise tool for dissecting the mechanisms by which these syllabic components are organized and articulated. This is crucial for understanding the sequential ordering within syllables, how each component influences the others, and the overall coordination requires for fluent speech production.

### The role of practice in speech production

Practice constitutes a central mechanism through which the human speech system refines its temporal and sequential organization. Repeated articulation of phonological sequences—whether words, syllables, or nonwords—enhances the fluency and stability of production by strengthening the mappings between abstract phonological representations and motor articulatory commands (Levelt, 1999; Dell et al., 1999; Guenther, 2016). From a cognitive perspective, practice promotes the automatization of speech planning processes, reducing the demands on working memory and executive control during articulation (Anderson, 1982; Logan, 1988). Empirical studies on repetition and nonword learning demonstrate that repeated exposure to novel phonological sequences improves recall accuracy and response speed, indicating more efficient serial ordering and reduced competition between phonological units (Gupta and MacWhinney, 1997; Acheson and MacDonald, 2009b). These practice-related improvements have been attributed to the gradual tuning of a speech production system, which becomes more adept at maintaining and manipulating sound-based representations through repeated production (Dell et al., 2000). At the neural level, practice facilitates procedural learning in cortico–basal ganglia–cerebellar circuits, which are known to support the transition from controlled to automatic stages of speech motor performance (Rizzolatti and Arbib, 1998; Guenther, 2016). In speech production research, the use of nonwords provides an ideal paradigm for examining practice effects, as it isolates phonological encoding and sequencing mechanisms from lexical-semantic influences. Repetition of nonwords thus serves not merely as a methodological device, but as a theoretical probe into how the speech production system consolidates new phonological patterns, adjusts timing, and refines serial order control through experience. Within this framework, the present study investigates how repeated nonword production in Korean engages practice-driven optimization of phonological encoding, testing whether serial ordering principles—phonological similarity, edge, and syllabic constraint—are dynamically modulated by experience-based learning processes.

### The current study

We used four-syllable CVC nonwords to balance theoretical sensitivity and task demands in testing serial order control. First, a four-item sequence provides clean leverage on the edge principle by yielding both primacy and recency positions plus two interior positions, allowing position-specific contrasts without the confounds that arise when “middle” collapses across too many slots (Page and Norris, 1998; Hurlstone et al., 2014). Second, four syllables sit comfortably within the core capacity of verbal working memory (≈4 ± 1 units), reducing floor/ceiling effects while preserving sufficient opportunity for adjacency-based interference (Botvinick and Plaut, 2006). Finally, nonword-repetition studies show that 3–4-syllable strings are optimal for revealing practice and sequencing effects in adults, maximizing diagnostic error variance without overwhelming articulation (Gathercole, 2006; Gupta and MacWhinney, 1997).

To investigate the impact of syllabic constraints on serial order control in speech production, particularly with Korean nonwords, this study drew on four established principles: phonological similarity, edge, and syllabic constraint. According to the phonological similarity principle, it was hypothesized that phonologically similar distractors presented before the production of target nonwords will lead to a higher incidence of speech errors compared to phonologically dissimilar distractors. This was because similar distractors activate comparable phonological nodes to the target nonwords, creating competition within the phonological output buffer and increasing the likelihood of incorrect phonological unit selection during speech production. Under the edge principle, we hypothesized that phonological units at the edges of nonwords (i.e., the first and last syllables) will be remembered and articulated more accurately, exhibiting fewer errors compared to internal units. This expectation stemmed from the well-documented primacy and recency effects, which suggest that items at the beginning and end of a sequence are typically better retained and produced with greater precision. Lastly, the syllabic constraint principle led us to predict that the rigid structural framework of CVC syllables in Korean will result in more consistent production of onset-vowel combinations than vowel-coda combinations. This consistency likely arises from the stronger linkage enforced by syllabic structuring rules, with the structural clarity of syllables in Korean—emphasizing distinct boundaries between onsets, nuclei, and codas—potentially influencing how these elements are integrated during speech production. By examining these hypotheses, this study aimed to enhance our understanding of how specific phonological and syllabic configurations affect serial order control in speech production.

## Method

### Participants

A total of 29 undergraduate and graduate students from Korea University participated in the current experiment and were compensated for their involvement. All participants were native speakers of Seoul Korean, thereby minimizing potential confounds from dialectal variation in both the perception and production of the stimuli. One participant who did not adhere to the experimental procedures was excluded from the final data analysis. Therefore, data from 28 participants (male: 17, female: 11) were utilized for analysis. The mean age of the participants was 26.3 years, with a range of 20 to 33 years. All participants provided informed consent prior to participating in the study. The study was conducted in accordance with the ethical principles outlined in the 1964 Declaration of Helsinki and was approved by the Institutional Review Board of Korea University (KUIRB-2024-0145-01).

### Materials

This study employed nonwords consisting of four Korean syllables with a C1V1C2 structure, which comprised an onset, nucleus, and coda. According to the phonological rules of Korean (onset + nucleus + coda), a theoretical maximum of 11,760 syllables can be generated through the combination of Korean consonants and vowels. However, analysis of the Korean Sejong National Corpus reveals that only 2,110 syllables (17.94%) are actively utilized, leaving 9,650 syllables unused in the Korean lexicon. To construct the 4-syllable targets and distractors, four syllables were randomly sampled among the remaining 9,650 syllables not found in the Korean Sejong National Corpus (Kang and Kim, 2009). A total of 1,350 nonword targets were constructed,^1^ ensuring that the combinational frequency of phonemes within the syllable and the frequency of adjacent syllables were both controlled at a low frequency. The similar distractors were created by interchanging one consonant or one vowel of adjacent syllables, while the dissimilar distractors shared no more than 50% of the phonemes of the targets (Nakayama and Saito, 2014). To illustrate, when the target was ‘졈푠컫젼’ (pronounced “jyeom pyon keot jyeon”), the similar distractor was ‘폄죤컫젼’ (pronounced “pyeom jyon keot jyeon”), in which the coda consonant of the first syllable was exchanged with the coda consonant of the second syllable. The similar distractors were made by exchanging two phonemes of the targets in either the onset, nucleus, or coda positions between the 1st and 2nd, 2nd and 3rd, or 3rd and 4th syllables. By contrast, a phonologically dissimilar distractor—for example, ‘텩잗뇬둉’ (tyeok-jat-nyon-dyong)—was paired with the target ‘횰뮴븆큽’ (hyol-myum-byut-keup) such that, when compared position by position across the four syllables (onset, vowel, coda), the two shared no more than half of their segments. In addition, 18 additional nonwords were used as filler targets. The stimuli were recorded by Korean text-to-speech software (Editor PANOPRETER).

### Operationalization of the four principles

Our experimental factors were explicitly chosen to operationalize three of the four well-established principles of serial order in speech production and verbal working memory. (1) Phonological similarity was manipulated by the distractor similarity factor (similar vs. dissimilar): similar distractors shared position-matched segments with the target (within onset, vowel, or coda), whereas dissimilar distractors shared ≤50% of segments across the four syllables. This tests the prediction that phonological overlap increases errors via competitive activation (Dell, 1986; Acheson and MacDonald, 2009b). (2) Edge effects were captured analytically by edge (1st–2nd and 3rd–4th vs. 2nd–3rd) and primacy (1st–2nd vs. 3rd–4th) contrasts, testing enhanced robustness for sequence boundaries (primacy/recency) relative to medial positions (Gupta, 2005; Gupta and Tisdale, 2009b). (3) Syllabic constraint was manipulated by the exchanged-within-syllable factor (onset vs. nucleus vs. coda) and examined with planned contrasts onset–rhyme (onset vs. nucleus+coda) and body–coda (onset+nucleus vs. coda). These contrasts probe position-specific cohesion within the Korean CVC syllable (Vousden et al., 2000). Consistent with our aims, the syllabic-structure contrasts (onset–rhyme; body–coda) were estimated within the similar-distractor condition, where structural relations are most diagnostic. All four principles are thus linked directly to (i) factorial manipulations (similarity; between-syllables; within-syllable) and (ii) prespecified coding of positional contrasts (edge; primacy) in the mixed-effects models.

### Design and procedure

**Figure 1:**
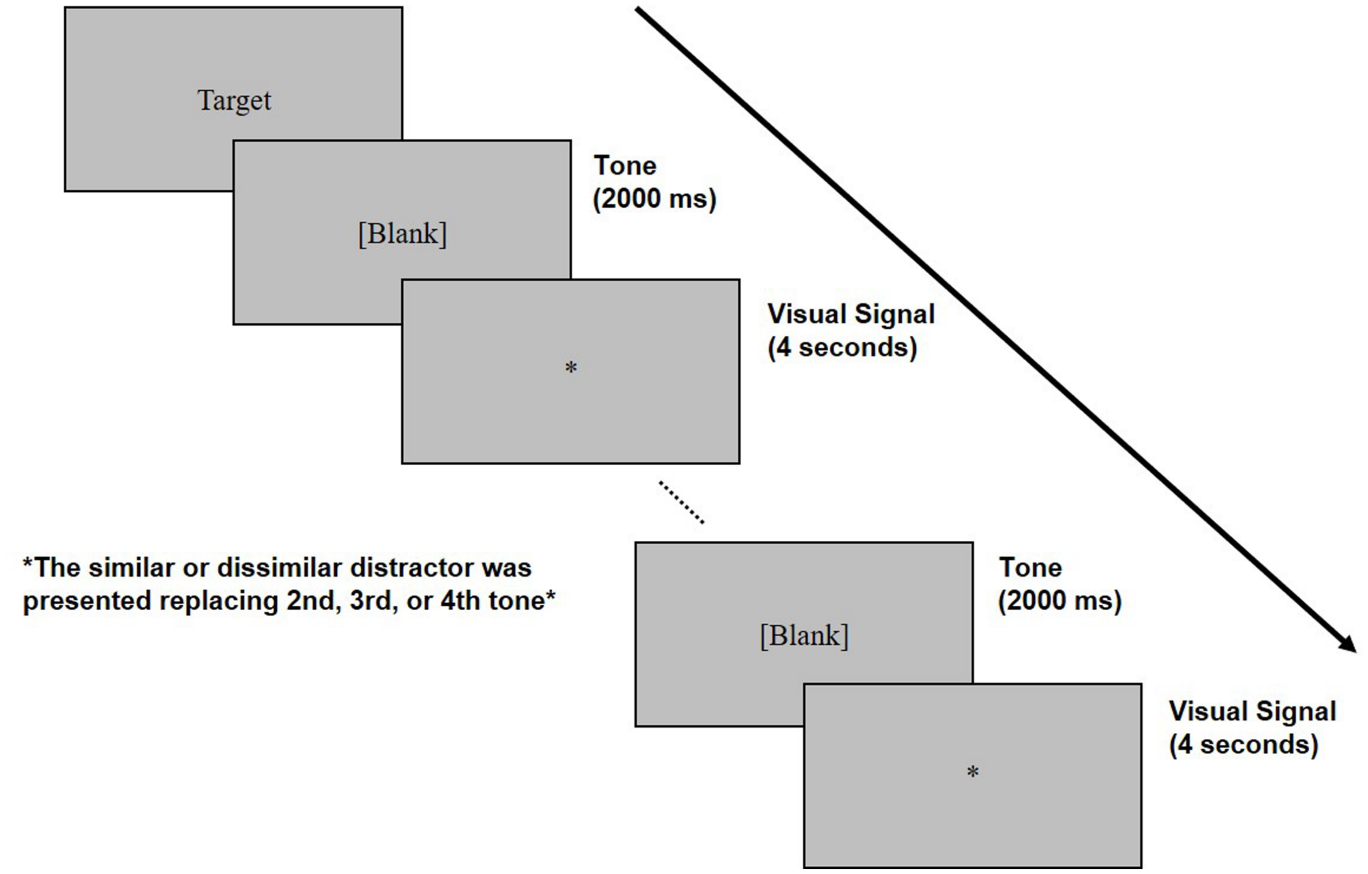
The experimental procedure of the Korean nonword speech induction technique.

Figure 1 presents the experimental procedure. Prior to the experiment, participants received instructions from the experimenter. At the beginning of each trial, the target nonword appeared on the screen, and participants produced it three times to confirm their pronunciation before proceeding. Then, participants needed to pronounce the target nonword whenever an asterisk mark appeared at the center of the computer screen. The asterisk mark was presented five times at a rate of one signal per 4 s, allowing participants to repeat the same target nonword five times per trial. A signal tone, presented at 440 Hz for 2000 ms, preceded the asterisk mark by 2000 ms. During a trial, an auditory distractor was randomly presented instead of the second, third, or fourth signal tone within a 2000 ms interval. All stimuli were presented via speakers (BITWAT studio-77), and the responses of participants were recorded using a Linear PCM recorder (SONY PCM-D50). The experiment consisted of 18 conditions with a 2 (distractor similarity: similar/dissimilar) × 3 (exchanged between syllable position: 1–2/2–3/3–4) × 3 (exchanged within syllable position: onset/nucleus/coda) × 3 (repetition: first/s/third participation) factorial design. The first three factors constructed 18 conditions, each included 5 trials, resulting in a total of 90 target trials. In each of the three repetition conditions, there were 108 test trials, including 18 filler trials. The trial order was pseudo-randomized, and participants performed seven practice trials before the test trials.

The current study employed a Latin square design to circumvent the length of time required for participants to complete the experimental procedure. Three distinct stimulus sets were created, each consisting of stimuli from all experimental conditions equally. Participants were randomly assigned to one of these sets and completed the experiment three times with the same set at an interval of either 3 or 4 days, corresponding to three repetition conditions.

### Statistical analyses

The current study investigated the effect of phonological similarity, syllabic structure, and repetition on speech accuracy in the repetitive nonword speech task. To examine these effects, logistic mixed-effect regressions were utilized since the dependent variable, speech accuracy, was binary. Specifically, responses were coded as 0 for errors and 1 for correct utterances. The fixed effects were orthogonalized except repetition variable^2^ and included phonological similarity (similar vs. dissimilar distractor), onset-rhyme (onset vs. nucleus and coda), body-coda (onset and nucleus vs. coda), edge (1st-2nd and 3rd-4th vs. 2nd-3rd), primacy (1st-2nd vs. 3rd-4th), repetition (first vs. second vs. third participation), and interaction effects (body-coda × edge, body-coda × primacy, and body-coda × repetition). The two factors of “onset-rhyme” and “body-coda” were limited to the phonologically similar distractor condition to reveal the syllabic structure in repetitive nonword speech, and the interactions between “body-coda” and other memorial variables (edge and primacy) were examined for syllabic constraint effects. The model includes all the fixed effects above and random effects for participants and items. The statistical analysis was conducted using the R software (R Core Team, 2012) and lme4 package (Bates and Maechler, 2010). The likelihood ratio tests were performed to obtain *p*-values for the effect under consideration by comparing the full model containing the effect with the model that does not include the effect.

## Result

### Preliminary analysis

All participant utterances were recorded and subsequently coded for errors by the experimenter. In addition to the experimenter’s assessment of speech errors, three independent raters who were native Korean speakers also confirmed these errors by referring to the voice records. Errors induced by the speech error technique were classified into two categories: incorrect errors and rhythm errors, according to Nakayama and Saito (2014). The former refers to phonological errors, such as the movement or substitution of phonemes, during target utterances, while the latter encompasses delayed responses, no responses, or responses during distractor presentation. As rhythm errors were not the focus of the present study, only incorrect errors were included in the main analysis (Nakayama and Saito, 2014). Moreover, only incorrect errors immediately after distractor presentation were tallied and analyzed, as was the case in previous research, while errors occurring before distractor presentation were not counted (Nakayama and Saito, 2014; Saito and Baddeley, 2004). In cases where raters disagreed on the classification of incorrect errors, only those errors agreed upon by more than two of the three raters were included in the main data analysis.

### Error categorization

The present study examined the types of speech errors that arose during Korean nonword production by referring to Nakayama and Saito (2014). Three types of speech errors were identified: phoneme movement, phoneme substitution, and pure deletion errors. Phoneme movement errors were further divided into exchange, anticipation, and perseveration errors. Exchange errors occurred when participants made errors in the form of a similar distractor, in which two phonemes triggered by the similar distractor were exchanged in the target utterance. Anticipation errors involved only the latter-to-earlier position movement, whereas perseveration errors involved only the earlier-to-latter position movement. Phoneme substitution errors occurred when at least one phoneme was replaced with a phoneme that was not in the targeted-position in the similar distractor. Pure deletion errors occurred when more than one phoneme in the targeted position was omitted. The qualitative categorization of speech errors is presented in Table 1, which indicates that more movement errors than deletion errors occurred and that the similar distractor induced more errors than the dissimilar distractor. Moreover, the error rate decreased with each successive repetition. These tendencies were analyzed using statistical techniques, which are described in the subsequent sections.

**Table 1:** A categorization of speech production errors: categorizing repetition, similar distractor, and dissimilar distractor as Rep., Sim., and Dis., respectively.

			Rep. 1		Rep. 2		Rep. 3	
			Sim.	Dis.	Sim.	Dis.	Sim.	Dis.
Movement								
Total			19	5	13	7	11	8
Exchange								
Syllable position								
	1–2	Onset	0	0	0	0	0	0
		Nucleus	2	0	0	0	0	0
		Coda	0	0	0	0	0	0
	2–3	Onset	0	0	0	0	0	1
		Nucleus	0	0	0	1	1	0
		Coda	0	0	0	0	0	0
	3–4	Onset	0	0	1	0	0	0
		Nucleus	0	0	0	0	0	1
		Coda	1	2	2	0	1	0
Anticipation								
Syllable position								
	1–2	Onset	0	0	0	0	0	0
		Nucleus	1	0	0	0	0	0
		Coda	3	1	0	0	1	1
	2–3	Onset	0	0	0	0	0	0
		Nucleus	0	0	0	0	0	1
		Coda	2	0	0	1	4	0
	3–4	Onset	0	0	0	0	0	0
		Nucleus	0	0	0	0	0	0
		Coda	2	0	1	3	1	0
Persiveration								
Syllable position								
	1–2	Onset	0	0	0	0	0	0
		Nucleus	2	0	0	0	0	0
		Coda	0	0	0	0	0	0
	2–3	Onset	0	0	0	0	0	1
		Nucleus	0	0	0	1	1	0
		Coda	0	0	0	0	0	0
	3–4	Onset	0	0	1	0	0	0
		Nucleus	0	0	0	0	0	1
		Coda	1	2	2	0	1	0
Substitution								
Total			22	12	23	11	18	7
Syllable position								
	1–2	Onset	3	0	1	0	0	0
		Nucleus	3	1	2	4	3	2
		Coda	1	2	3	0	2	0
	2–3	Onset	2	1	0	0	0	1
		Nucleus	2	5	3	3	3	1
		Coda	5	1	2	0	1	1
	3–4	Onset	0	0	4	1	2	0
		Nucleus	2	0	2	1	4	2
		Coda	4	2	6	2	3	0
Pure deletion (incomplete)			2	3	5	1	3	0
Correct			7,517	7,540	7,519	7,541	7,528	7,545
# of trials			7,560					

### Error analysis

**Figure 2:**
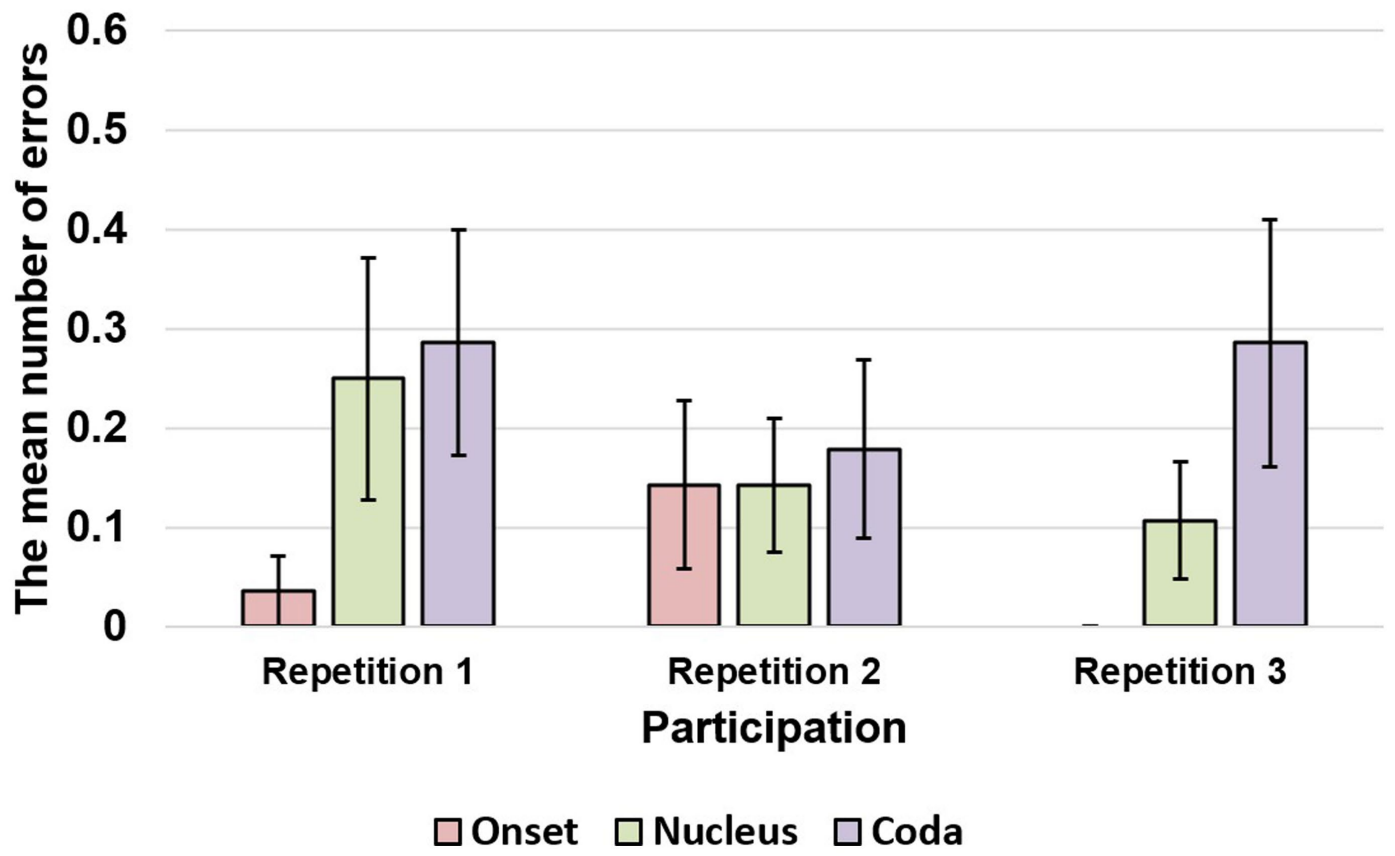
The number of errors in onset, nucleus, and coda within a syllable as speech practices proceeded. The line in the bar indicates the range of standard error.

The present study employed logistic mixed-effect regressions to examine the effects of various factors on Korean nonword speech errors. Results showed that the phonological similarity of the distractor had a significant main effect [ $\chi^{2}$ (13) = 34.640, *p* < 0.001], with more errors occurring in the similar distractor condition compared to the dissimilar distractor condition. The onset-rhyme main effect was not significant [ $\chi^{2}$ (13) = 16.866, *p* = 0.206], meaning no difference of the number of errors between the onset and rhyme positions. Furthermore, the body-coda main effect was significant [ $\chi^{2}$ (12) = 55.337, *p* < 0.001], indicating that more errors were observed in the coda position than in the body. The repetition main effect was also significant [ $\chi^{2}$ (13) = 44.576, *p* < 0.001], suggesting that errors decreased as participants repeated the task. Additionally, a significant interaction between body-coda and repetition was found [ $\chi^{2}$ (23) = 61.436, *p* < 0.001], with body errors decreasing as repetition proceeded and coda errors remaining constant. See Figure 2 for the graphical results.

**Figure 3:**
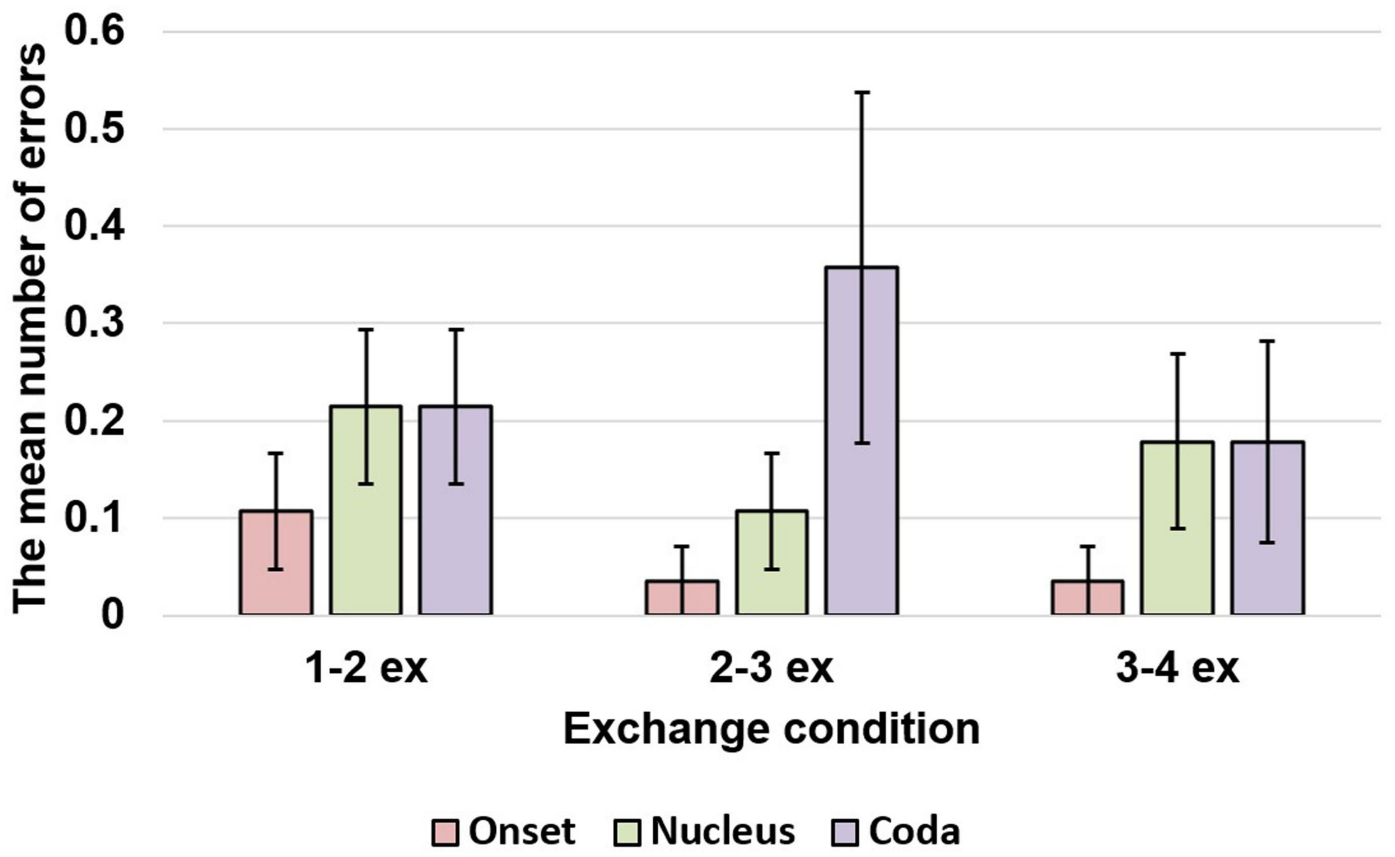
The number of errors in onset, nucleus, and coda within a syllable in exchanged conditions. The line in the bar indicates the range of standard error.

Moreover, the study investigated aspects of serial order control and found a significant interaction effect between body-coda and edge [ $\chi^{2}$ (19) = 51.862, *p* < 0.001], even though the main effect of edge was not significant [ $\chi^{2}$ (8) = 2.702, *p* = 0.952]. The post-hoc test for the significant interaction effect indicates more coda errors in the 2–3 exchanges than in the 1–2 and 3–4 exchanges [ $\chi^{2}$ (7) = 14.933, *p* = 0.037], while body errors remained unchanged [ $\chi^{2}$ (7) = 7.881, *p* = 0.343]. The main effect of primacy gradient was not significant [ $\chi^{2}$ (8) = 4.048, *p* = 0.858], but a significant interaction effect was observed between body-coda and primacy gradient [ $\chi^{2}$ (19) = 56.875, *p* < 0.001]. However, the post-hoc test for the significant interaction did not reveal any significant primacy effects in both body and coda [ $\chi^{2}$ (7) = 1.911, *p* = 0.965 for body; $\chi^{2}$ (7) = 6.957, *p* = 0.433 for coda]. See Figure 3 for the graphical results.

In summary, the fixed effects of “phonological similarity”, “body-coda”, “repetition”, “body-coda × edge”, “body-coda × primacy gradient”, and “body-coda × repetition” were significant in the likelihood tests, while “onset-rhyme”, “edge”, and “repetition” were not significant. See Table 2 for a summary of the results.

**Table 2:** Results of logistic mixed-effects regression analysis in experiment.

Groups		Variance	SD	Correlation					
*Random effects*									
Item	(Intercept)	1.06 $e^{-04}$	0.010						
	Similarity	4.09 $e^{-05}$	0.006	−0.57					
	Onset-Rhyme	5.98 $e^{-06}$	0.002	−0.13	0.89				
	Body-Coda	8.02 $e^{-05}$	0.009	−0.88	0.89	0.58			
	Repetition	4.78 $e^{-05}$	0.007	−0.92	0.85	0.51	1		
Subject	(Intercept)	4.20 $e^{-05}$	0.006						
	Similarity	3.01 $e^{-05}$	0.005	1.00					
	Onset-Rhyme	5.56 $e^{-06}$	0.002	0.54	0.54				
	Body-Coda	2.82 $e^{-05}$	0.005	0.50	0.50	−0.46			
	Edge	3.38 $e^{-05}$	0.006	0.13	0.13	−0.73	0.92		
	Primacy gradient	4.80 $e^{-06}$	0.002	0.85	0.85	0.34	0.60	0.38	
	Repetition	1.47 $e^{-06}$	0.001	−0.22	−0.22	−0.93	0.74	0.93	0.03

	Estimate	SE	*t*-value	$\chi^{2}$	Pr( $\chi^{2}$ )
*Fixed effects*					
(Intercept)	0.990	0.003	318.52		
Similarity	−0.002	0.002	−1.091	33.16	<0.01
Onset-Rhyme	−0.002	0.001	−1.613	13.64	>0.1
Body-Coda	0.002	0.002	1.035	57.26	<0.01
Edge	−0.001	0.002	−0.116	22.63	<0.01
Primacy gradient	0.002	0.003	0.860	1.76	>0.1
Repetition	0.001	0.001	0.916	27.95	<0.01
Body-Coda × Edge	−0.002	0.001	−1.254	70.30	<0.01
Body-Coda × Primacy gradient	0.001	0.002	0.134	59.17	<0.01
Body-Coda × repetition	−0.001	0.001	−1.514	69.90	<0.01

## Discussion

We used a speech-error induction paradigm to test how Korean’s syllabic organization shapes serial control during production. We treat “serial order” within a hierarchical framework, where syllables and their subsyllabic parts are not just arranged left to right. Instead, their temporal selection is conditioned by a Korean-specific organization in which the body (onset plus vowel) and the coda play distinct roles (Lashley, 1951; Yi, 1998; Verdonschot et al., 2021; Li et al., 2022). With unfamiliar four-syllable CVC nonwords, we observed robust phonological-similarity effects in the speech errors, which were induced for phonemes movements in adjacent syllable positions, indicating competition among concurrently active phonological representations and greater interference for adjacent selections—both signatures of order coding operating within a higher-level structure (Acheson and MacDonald, 2009b; Botvinick and Plaut, 2006).

### The robustness of the findings in speech error patterns

Although the speech error induction technique resulted in a relatively low incidence of errors, previous studies, such as those by Nakayama and Saito (2014), have demonstrated that even a small number of errors can yield significant insights into the cognitive processes underlying speech production. Nakayama and Saito (2014) showed that the small number of errors made during the repetitive production of words and nonwords could substantiate the mechanisms of serial order in speech, highlighting the utility of such errors in revealing the complexities of speech dynamics. This aligns with findings from earlier studies like those of Saito and Baddeley (2004), where the consistency of error patterns, especially those related to phonological similarity effects, underscored the reliability and validity of the speech error induction technique in exploring cognitive aspects of speech production.

Despite the low frequency of errors, the current study was able to replicate these phonological similarity effects successfully, confirming the method’s effectiveness and the generalizability of these findings. The robustness of the speech error induction technique is evidenced by its ability to consistently provoke specific types of errors across different iterations and settings, thereby allowing researchers to draw reliable conclusions about the underlying structures and processes of speech production. This ability to induce and then analyze even a minimal number of speech errors provides a significant result for researchers, offering a refined lens through which the language processing can be examined. Consequently, the findings from the current study not only corroborate the effectiveness of using induced errors to understand speech production but also contribute to a broader understanding of how phonological information is organized and manipulated within the cognitive framework of language use.

### Examination of serial order control using Korean syllabic constraint

The current study examined serial order control in speech through the lens of Korean syllable structure. Whereas English is often modeled in terms of onset–rhyme organization (Kubozono, 1991; Treiman, 1989), Korean appears to privilege a body–coda partition in which the onset–vowel “body” forms a tighter unit than the vowel-coda “rhyme” (Lee and Nam, 2020). Building on this typological contrast, we tested whether error patterns in Korean nonword production reflect this hierarchy. As predicted, phonologically similar distractors produced more errors than dissimilar ones, corroborating the phonological similarity effect in production. Crucially, errors concentrated in codas relative to body elements, indicating weaker serial stability for vowel–coda pairings than for onset–vowel pairings and supporting a syllable-internal asymmetry consistent with Korean body–coda organization. This asymmetry dovetails with independent evidence for syllable- and CV-body–based preparation in Korean production tasks (Verdonschot et al., 2021; Li et al., 2022). At the multi-syllable level, contrasts between adjacent positions revealed robust temporal-distance effects without a reliable edge advantage, suggesting that local adjacency—rather than global boundary status—was the primary determinant of order stability in this paradigm (Gupta, 2005; Gupta and Tisdale, 2009b). A residual primacy tilt emerged for body components but not for codas, further implying position-sensitive coupling within the syllable. In short, the loci of serial errors in Korean are best explained not by a flat left-to-right sequence of segments but by serial selection operating within a language-specific syllabic hierarchy.

These findings suggest the intricate ways in which syllabic structure and serial order control interplay in the production of speech. The evidence that Korean syllabic characteristics − specifically, the comparative robustness of onset-nucleus structures over codas − shape error patterns in speech production highlights the critical need to consider language − specific phonological rules when studying linguistic processing.

### The syllabic constraint effect on speech error changes over the repetition of nonword speech production

The present study examined the impact of syllabic constraints, specifically focusing on the body and coda structures, on the control of serial order during the production of nonword speech, while also evaluating how established speech practices influence this relationship. The findings demonstrated a significant interaction between these syllabic constraints and repetition frequency, alongside a prominent main effect of repetition. This suggests that the structured nature of Korean syllables, particularly the integration of body and coda, plays a critical role in facilitating the learning of new sequences and in predicting consistent phonological representations as speech practices are repeated and refined. Such results are indicative of a systematic phonological processing mechanism that becomes more efficient with practice, consistent with predictive-monitoring accounts in which internal error correction reduces uncertainty across repeated sequences under distractor interference (Teghipco et al., 2023).

Furthermore, the current study sheds light on the transfer of phonological knowledge from familiar word syllables to unfamiliar nonword constructs, echoing phenomena observed in other cognitive domains such as motor sequence learning and second language acquisition (Müssgens and Ullén, 2015; Flege and Davidian, 1984). This pattern of knowledge transfer suggests that the cognitive strategies honed during first language acquisition are not only retained but also reactivated and effectively applied during second language speech production. The study aligns with previous research which shows that speakers who lack certain phonological features in their native language often reproduce these patterns in their acquisition of a second language (e.g., Macken and Ferguson, 1981), highlighting a universal aspect of phonological learning.

### The influence of the syllabic constraint on the phonological encoding

The present study provides further support for the importance of syllables in Korean speech production as the phonological unit, which has been reported in previous studies (Verdonschot et al., 2021). According to Verdonschot et al. (2021), the syllable is a proximate unit in Korean for phonological encoding due to its less complexity, with clearer syllable boundaries than European languages such as Dutch and English. This unique characteristic of Korean syllables underpins a clear representation of syllables within words and nonwords, leading to strong syllabic constraints in phonological encoding. The current study demonstrates an evident syllabic constraint effect in serial order control for speech production, revealing a differential effect of serial order control for onset and coda positions in syllables, indicating that the onset is more tightly chained with the nucleus than the coda. It suggests that each position within syllables may be represented with different strengths due to the syllabic constraint during phonological encoding.

The theoretical models of speech production have previously explained how we organize speech (e.g., Caramazza, 1997; Levelt et al., 1999). For instance, Levelt et al. (1999) proposed a model to describe phonological encoding in speech production, which assumes two types of information: the metrical frame and the phonological units. The metrical frame indicates information on the length of words in a unit of syllables (i.e., the number of syllables) and the position of stress in words. In contrast, phonological units denote the selected units at the early stage to prepare the metrical frame. In light of the present study, the findings suggest that the syllabic constraint affects the formation of phonological units for phonological encoding and their subsequent processing for speech production.

Thus, the existence of the syllabic constraint on phonological encoding highlights the need to consider the impact of language-specific features on speech production models. For example, Roelofs (2015) revised the speech model to account for findings using languages with certain morphological complexity (O’Séaghdha et al., 2010; Verdonschot et al., 2011). Previous studies have reported that the proximate unit for phonological encoding varies across languages, such as phonemes for Dutch and English, syllables for Chinese, and morae for Japanese (O’Séaghdha, 2015; Roelofs, 2015). This suggests the need to reconsider the proposed speech production models to accommodate other languages, including Korean. Additionally, the present study indicates that all subcomponents of the proximate unit, i.e., onset or coda in syllables, do not have the same status of representation for phonological encoding since they are affected by the syllabic constraint. The auditory distractors in the present study resulted in more errors at the coda than at the onset in syllables, suggesting weaker representation at the coda than at the onset as the subcomponent of syllables (the proximate unit).

### Limitations

Although the present study yielded valuable insights into serial order control in Korean nonword production, several limitations constrain the generalizability of our findings. First, the sample showed a gender imbalance (17 men, 11 women), which may have subtly affected speech error rates or their modulation by experimental conditions. While contemporary models of phonological encoding do not posit systematic gender effects, previous studies have reported sex-related differences in speech motor coordination, articulatory timing, and variability, suggesting that production mechanisms may diverge across genders. Accordingly, we cannot fully rule out the influence of sample composition on the observed outcomes.

Second, the modest sample size (*n* = 28) limited statistical power, particularly for detecting smaller effects or higher-order interactions in the mixed-effects regression analyses. This limitation heightens the likelihood of Type II errors and necessitates cautious interpretation of non-significant results.

Third, language processing is known to be asymmetrically organized across the hemispheres (Kim et al., 2022a; Kim and Nam, 2024), with the left hemisphere playing a dominant role in phonological planning and articulation (Poldrack et al., 1999). Speech production may therefore engage lateralized neural dynamics that vary across individuals. Prior research has demonstrated that interhemispheric facilitation enhances familiar word processing (Kim et al., 2022b) but that the pattern of hemispheric interaction depends on temporal spacing between visual words (Kim and Nam, 2023a, 2023b, 2024, 2025a) and on semantic context (Kim and Nam, 2023b, 2025b). Hence, individual differences in hemispheric asymmetry for phonological encoding could contribute to variability in speech errors during nonword production, particularly given the left hemisphere’s specialization for phonological computation.

Fourth, cognitive and propensity-related factors may further modulate speech production. Kim et al. (2025) found that risk-taking propensity influences lexical decision performance, with high-risk individuals showing slower and less accurate recognition and greater response bias toward both words and nonwords. Such findings imply that individual cognitive styles and decision tendencies could shape phonological processing under time-constrained speech production demands.

To address these limitations, future research should adopt a multi-pronged approach. Specifically, studies should (i) recruit gender-balanced and demographically diverse samples to ensure representativeness and assess gender-specific patterns in phonological encoding and error production; (ii) substantially increase sample size to achieve sufficient power for detecting subtle main effects and higher-order interactions, while accommodating maximal random-effects structures; (iii) incorporate neurocognitive and neuroimaging measures to directly assess hemispheric dynamics during nonword production, enabling precise mapping between behavioral variability and lateralized processing; and (iv) systematically evaluate individual cognitive traits—such as risk-taking propensity, executive control, and working memory capacity—as potential moderators of speech production performance. Such methodological refinements will advance a more mechanistic and generalizable understanding of how cognitive, neural, and individual factors jointly shape phonological sequencing and speech error dynamics.

## Conclusion

The present study aimed to investigate the impact of syllabic constraints on serial order control during Korean nonword speech production using the speech error induction technique. The study had three main objectives. Firstly, it aimed to examine the phonological syllabic constraints (body + coda) in speech production by analyzing the error rates with onset- and nucleus-exchanged distractors in comparison to coda-exchanged distractors. Secondly, the study sought to identify the effects of syllabic constraints on serial order control by comparing the edge effect in coda and body. Lastly, nonword speech practice was conducted to investigate the strong syllabic constraints in syllables within nonwords and how the practice influences error reduction in body relative to coda. The results suggest the significant impact of syllabic constraints on serial order control in speech production. Specifically, the study found that the coda-exchanged distractors elicited more errors compared to onset- and nucleus-exchanged distractors. Moreover, the edge effect was more prominent in the coda compared to the body, indicating the effect of syllabic constraints on serial order control. The findings of the nonword speech practice indicated a strong influence of syllabic constraints in syllables within nonwords. Overall, the study highlights the crucial role of syllabic constraints in the organization of speech production in Korean.

## Data Availability

The datasets presented in this study can be found in online repositories. The names of the repository/repositories and accession number(s) can be found in the article/supplementary material.

## Footnotes

1 Given the breadth of phonological alternations in Korean, exhaustively controlling all possible variants in a nonword paradigm is not feasible. To preserve ecological validity, we therefore generated a large, heterogeneous set of nonwords via random recombination, ensuring that participants produced a broad range of forms rather than a narrow, hand-selected list.

2 The fixed-effect variable “repetition” in the current study has three levels and was not coded orthogonally to test its effect. Generally, when fixed-effect variables in regression analysis are not orthogonal, there may be a risk of multicollinearity among them, which can distort the results. However, the multicollinearity check for the model in this study showed that the VIP (variance inflation factor) for each fixed-effect variable was below 5, indicating that there was no multicollinearity problem among the fixed-effect variables (similarity = 1.256, onset-rhyme = 1.306, body-coda = 3.145, edge = 1.153, primacy = 1.153, repetition = 1.068, body-coda × edge = 1.131, body-coda × primacy = 1.140, body-coda × repetition = 2.673).
